# Clinical comparative study on Nitrous Oxide inhalation versus intravenous propofol and Midazolam sedation in Transnasal Gastroscopy

**DOI:** 10.12669/pjms.334.12290

**Published:** 2017

**Authors:** Zhou Xiaoqian, Zhang Tao, Luo Bingsong, Li Jing, Deng Yu, Zhong Weilan

**Affiliations:** 1 Dr. Zhou Xiaoqian, Department of Gastroenterology, The First People’s Hospital of Gui Yang, Gui Zhou Province, China; 2 Dr. Zhang Tao, Department of Gastroenterology, The First People’s Hospital of Gui Yang, Gui Zhou Province, China; 3 Dr. Luo Bingsong, Department of Gastroenterology, The First People’s Hospital of Gui Yang, Gui Zhou Province, China; 4 Dr. Li Jing, Department of Gastroenterology, The First People’s Hospital of Gui Yang, Gui Zhou Province, China; 5 Deng Yu, Department of Gastroenterology, The First People’s Hospital of Gui Yang, Gui Zhou Province, China; 6 Zhong Weilan, Nursing Department, The First People’s Hospital of Gui Yang, Gui Zhou Province, China

**Keywords:** Intravenous propofol and midazolam sedation, Nitrous oxide inhalation, Transnasal gastroscopy

## Abstract

**Objective:**

To investigate the Clinical practice value of nitrous oxide inhalation and intravenous propofol and midazolam sedation in transnasal gastroscopy.

**Method:**

From December 2012 to April 2014, two hundred patients receiving painless transnasal gastroscopy on a voluntary basis were selected in Endoscopy center, The First People’s Hospital of GuiYang. Patients were divided into two groups: Group-1 consisted of one hundred patients sedated by nitrous oxide inhalation and Group-2 consisted of one hundred patients sedated by intravenous propofol and midazolam. Patients were then examined by transnasal gastroscopy. Patient blood pressure, heart rate, pulse rate and oxygen saturation before, during and after gastroscopy were recorded for both groups. The duration of the gastroscopy and the time of awakening were also recorded. After examination, the patients were asked to assess the level of discomfort experiences during the gastroscopy procedure.

**Results:**

All patients successfully underwent the transnasal gastroscopy. There were 57 males and 43 females in the nitrous oxide inhalation group with an average age of 43.11±8.27 years. The average duration of examination and time of awaking in the nitrous oxide inhalation group was of 152.7±9.80 secs and 50±7.89 secs respectively. For the intravenous propofol and midazolam sedation group, there were 53 males and 47 females with an average age of 41.26±7.98 years. The average duration of examination and time of awaking in the intravenous propofol and midazolam sedation group was of 149.07±10.25 seconds and 390±20.89^#^ seconds respectively. The two groups showed no significant difference in the duration of examination. There was no difference in the age or sex. The former had a less significant impact on heart rate, oxygen saturation and blood pressure, while the intravenous propofol and midazolam sedation decreased blood pressure dramatically and this effect persisted after examination.

**Conclusion:**

Nitrous oxide inhalation has higher safety and tolerance with a brighter application prospect for transnasal gastroscopy.

## INTRODUCTION

Painless endoscopy is now widely used in the clinic. However, the most common intravenous anaesthetics inhibit respiration and circulation, with intravenous anesthesia usually requiring continual assessment and monitoring by an anaesthetist. Nitrous oxide/oxygen inhalation sedation has proved to be a safe and effective approach for controlling pain and anxiety. Because of simplicity, convenience, less adverse events and lower costs, nitrous oxide/oxygen inhalation sedation has become the common sedation technique for pains induced by abortion and dental treatment.^1^-^3^ We compared the effects of nitrous oxide inhalation and intravenous propofol and midazolam sedation in transnasal gastroscopy. The safety and effectiveness of the two kinds of anaesthesia techniques were compared.

## METHODS

From December 2012 to April 2014, two hundred patients receiving painless transnasal gastroscopy on a voluntary basis were selected in Endoscopy center, The First People’s Hospital of GuiYang. There were 57 males and 43 females in the nitrous oxide inhalation group, who were aged 20-78 years old with an average of 43.11±8.27 years. For the intravenous propofol and midazolam sedation group, there were 53 males and 47 females, aged 8-74 years old with an average of 41.26±7.98 years. The two groups showed no significant difference in the duration of examination (p>0.05). The following patients were excluded: allergy to soybeans, nitrites, egg products and propofol; combined with heart diseases, which were symptomatic upon transnasal gastroscopy; contraindicated for nitrous oxide inhalation and transnasal gastroscopy; planning pregnancy or during the first 3 months of pregnancy; comatous state, within one week after pneumoencephalography, middle ear diseases, aerothorax, pulmonary cystic fibrosis, bowel obstruction, having received nasal sinus surgery or nasal septum surgery, oxygen saturation below 95%, systolic pressure below 90mmHg. The informed consent was obtained from all patients. The experiment was approved by the ethics committee of the First People’s Hospital of Guiyang.

AII 5000C dental nitrous oxide/oxygen sedation system (Shenzhen Anbao Technology Company), electronic transnasal gastroscope(FujinonEG-530N), multi-parameter monitor (SPR9000), propofotl (Fresenius Kabi Deutschland), midazolam(Jiang Su En Hua pharmaceutical Co. Ltd).

All patients were fasted for over 12 hour before examination. At 10 minutes before transnasal gastroscopy, 10ml of lidocaine plasmagel was administered bucally. For the intravenous propofol and midazolam sedation group, an oxygen mask was used along with intravenous injection of propofol and midazolam. The patients’ response was observed during administration. Transnasal gastroscopy was performed when the patients fell into sleep with smooth breathing and disappearance of eyeflash reflexes. The propofol dose was 1.5-2mg/kg, and midazolam dose was 2mg/kg. In case of dramatic decline of blood pressure or bradycardia (heart rate<50 beats/min), 5mg ephedrine or 0.25mg atropine was administered intravenously. No more anesthetics were injected after examination. When the patients had completely awoken from anesthesia, they were further observed for 10 minutes and then discharged. For the nitrous oxide inhalation group, the nitrous oxide/oxygen mixture was inhaled. The concentration of nitrous oxide increased from 0 to 50-70%. After reaching the expected anesthetic effect, transnasal gastroscopy was performed. When the patients had completely awoken from anesthesia, they were further observed for five minutes and then discharged.

### Evaluation criteria for the anesthetic effect

Visual analog scale (VAS): 0 point was defined as no pain or any sensation; 1-3 point, mild pain, but not to the extent of interfering with daily life activities and work; 7-10 point, severe pain that interfered with daily life activities and work.

### Observation indicators

Blood pressure, heart rate, pulse rates and oxygen saturation before, during and after gastroscopy were recorded in the two groups. The duration of gastroscopy operation and awakening time were also recorded. After examination, the patients were surveyed by questionnaires and inquired whether they were willing to receive the transnasal gastroscopy again and whether they were satisfactory with the transnasal gastroscopy.

### Statistical analysis

Measurements were expressed as Mean±SD. All statistical analyses were performed using the SPSS 12 software. The means of the two samples were compared with t-test. Counts were compared using the χ^2^ test, with the significance level set as α=0.05.

## RESULTS

Both groups showed no significant difference in the gender ratio and average age (P>0.05). Table-I

**Table-I:** Comparation of gender ratio and age.

*Group*	*Gender ratio (male/female)*	*Age (years)*
1	57/43	43.11±8.27
2	53/47	41.26±7.98

Before gastroscopic examination, the two groups had no significant difference in blood pressure, heart rate and oxygen saturation. Table-II. As compared with the levels before gastroscopic examination, the nitrous oxide inhalation group during gastroscopic examination showed a mild increase of blood pressure and heart rate, while the intravenous propofol and midazolam sedation group during gastroscopic examination showed a significant decrease of blood pressure and heart rate. Moreover, blood pressure and heart rate decreased less dramatically in the nitrous oxide inhalation group as compared with the intravenous propofol and midazolam sedation group during gastroscopic examination. After gastroscopic examination, blood pressure and heart rate of the nitrous oxide inhalation group recovered quickly and they were not significantly different from the levels before gastroscopic examination. However, after gastroscopic examination, blood pressure of the intravenous propofol and midazolam sedation group was still much lower than that before gastroscopic examination. The two groups showed no significant difference in oxygen saturation and heart rate before or after gastroscopic examination.

**Table-II:** Comparation of pressure, heart rate and Oxygen saturation.

*Group*	*Systolic pressure (mmHg)*			*Diastolic pressure (mmHg)*		

	*Before gastroscopic examination*	*During gastroscopic examination*	*After gastroscopic examination*	*Before gastroscopic examination*	*During gastroscopic examination*	*After gastroscopic examination*
1	126.75±12.69	127.07±12.09	124.20±11.73	77.32±9.36	78.01±6.89	78.05±7.24
2	123.83±15.55	116.07±9.37*	117.66±10.42*	78.35±7.45	75.18±7.97*	75.92±6.79*

	***Heart rate (beats/min)***			***Oxygen saturation %***		

	***Before gastroscopic examination***	***During gastroscopic examination***	***After gastroscopic examination***	***Before gastroscopic examination***	***During gastroscopic examination***	***After gastroscopic examination***

1	84.3±10.49	85.49±8.81	82.54±9.27	97.28±0.01	97.31±0.02	97.1±0.01
2	86.06±7.79	83.01±8.55*	85.96±6.89	97.44±0.01	94.96±0.02*	97.54±0.01

* P<0.05: compared with the level before examination.

### Comparison of effectiveness

The duration of gastroscopy was 77-240s in the nitrous oxide inhalation group with an average of 152.7±9.80s. Table-III. The duration of gastroscopy was 60-303s in the intravenous propofol and midazolam sedation group with an average of 149.07±10.25s. The two groups had no significant difference in the duration of gastroscopy (p>0.05). The VAS score was 2.01±0.01 in the nitrous oxide inhalation group and 2.32±0.02 in the intravenous propofol and midazolam sedation group, also indicating no significant difference (p>0.05). The awakening time was 30-85s in the nitrous oxide inhalation group with an average of 50±7.89s, and 350-487s in the intravenous propofol and midazolam sedation group with an average of 390±20.89s. There was significant difference in awakening time (p<0.05).

**Table-III:** Comparison of duration of gastroscopy, awakening time and VAS score.

*Group*	*Duration of gastroscopy (s)*	*Awakening time (s)*	*VAS score*
1	152.7±9.80	50±7.89	2.01±0.01
2	149.07±10.25	390±20.89#	2.32±0.02

#P<0.05: Compared with the nitrous oxide inhalation group.

### Questionnaire survey

In the nitrous oxide inhalation group, 95% (95/100) of the patients were willing to receive transnasal gastroscopy for a second time; in the intravenous propofol and midazolam sedation group, 96% (96/100) of the patients were willing to receive transnasal gastroscopy for a second time. The satisfactory rate was 96% (96/100) in the nitrous oxide inhalation group and 97% (97/100) in the intravenous propofol and midazolam sedation group. There was no significant difference in the satisfactory rate (P>0.05).

## DISCUSSION

Transnasal gastroscopy is a safe and feasible examination technique, which is widely accepted among the patients due to its high tolerance.^4^,^5^ Propofol is a short-acting intravenous anesthetic and has a short awakening time. It can properly control patients’ anxiety though it suppresses respiration transiently, with considerable cardiovascular suppression. Nitrous oxide is a colourless and mildly sweet inorganic gas with stable chemical properties, non-in flammability, and non-explosiveness; it can support combustion and inhibit the release of excitatory substances of the central nervous system, as well as the propagation of nerve impulses. The nitrous oxide works by changing the permeability of the ion channels. The peak clinical effect usually occurs 3-5 minutes after inhalation and 99% of the inhaled nitrous oxide is excreted quickly from the lung without biotransformation. The nitrous oxide can also diffuse quickly in the blood. Because of these advantages, nitrous oxide is widely used in dental surgeries and childbirth, as well as transnasal gastroscopy.^6^-^8^ However, the effects of nitrous oxide inhalation and intravenous propofol and midazolam sedation in transnasal gastroscopy were compared in only a limited number of researches.

We compared the safety and effectiveness of nitrous oxide inhalation and intravenous propofol and midazolam sedation in transnasal gastroscopy. The result showed that the former had a less significant impact on heart rate, oxygen saturation and blood pressure, while the intravenous propofol and midazolam sedation decreased blood pressure dramatically and this effect persisted after examination. The two groups had similar duration of gastroscopy examination and comparable VAS scores. The two anesthesia techniques can achieve similar sedation and anesthetic effects. Patients in the nitrous oxide inhalation group awoke more quickly from anesthesia and the difference was significant compared with the intravenous propofol and midazolam sedation group. The above results indicated that the anesthetic effect of nitrous oxide disappeared more rapidly after examination.

To conclude, nitrous oxide inhalation has the following advantages compared with the intravenous propofol and midazolam sedation:

1)The operation is easily performed and ordinary physicians or nurses are qualified after training.
2)The respiratory and circulation functions are maintained stable during gastroscopy. The anesthesia is safe and the patients can awake quickly from anesthesia, thus reducing the time of hospitalization.
3)The patients stay conscious during examination and the pharyngeal reflex is present, which reduces the risk of aspiration. The patients report similar satisfaction degree with either anesthesia technique.

Therefore, nitrous oxide inhalation has higher safety and tolerance with a brighter application prospect for transnasal gastroscopy.

### Author’s Contribution

**ZXQ** conceived, designed and did statistical analysis, wrote manuscript & editing of manuscript.

**ZT, LBS and LJ** collected data.

**DY and ZWL** reviewed and approved final manuscript.
